# A systemic look at pancreatic cancer patients: Predicting metastasis by studying the liver

**DOI:** 10.1038/s41392-024-01964-4

**Published:** 2024-09-27

**Authors:** Susanne Roth, Christoph Michalski, Jörg D. Hoheisel

**Affiliations:** 1 Department of General Surgery, University Hospital Heidelberg, Heidelberg, Germany; 2 Division of Functional Genome Analysis, German Cancer Research Center (DKFZ), Heidelberg, Germany

**Keywords:** Metastasis, Tumour biomarkers

In a recent paper published in *Nature Medicine*, Bojmar et al. describe an elaborate effort toward predicting the metastatic progress of pancreatic ductal adenocarcinoma (PDAC) after primary tumour resection.^1^ Instead of investigating the tumour, however, they studied several molecular, cellular, and metabolic features in biopsy samples from the seemingly still unaffected liver, which were collected during resection of the primary tumour, demonstrating the large and still neglected biomedical potential of looking at cancer in a systemic manner.

**Fig. 1:**
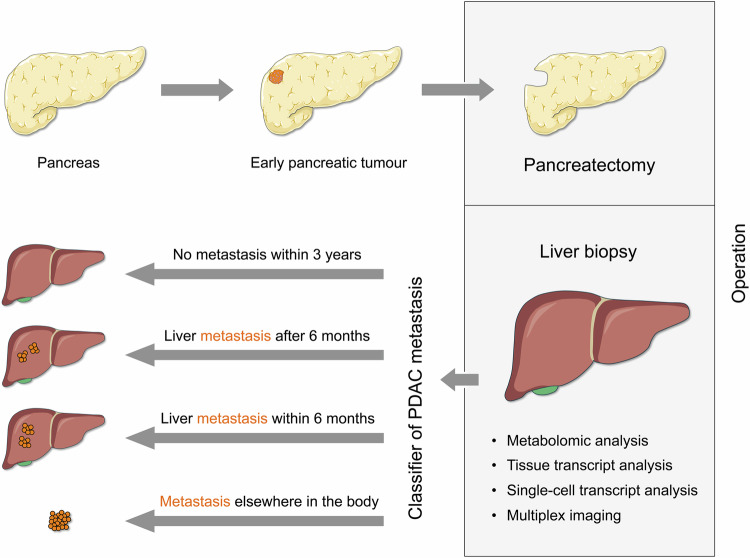
Schematic representation of the patient classification process. During the resection of primary PDAC tumours in the pancreas, liver biopsies were taken and analysed. On the basis of the liver data, classifiers could be created that allowed predicting time and location of metastasis

PDAC is a cancer with a dismal prognosis; mortality is close to incidence and untreated patients survive few months only. The best therapeutic option is resection of the cancerous part of the organ. However, only about 20% of patients are eligible for this. And even then, patients usually experience recurrence within relatively short periods of time. Bojmar and colleagues looked for means to classify the metastatic risk of such patients at the time of operation and predict both the timing and location of metastasis. Rather than focussing on the tumour, however, they actually analysed a series of different parameters in the pre-metastatic liver tissue, from which a biopsy was taken during pancreatectomy. Besides revealing interesting biological effects that may contribute to cancer growth and dissemination, they were able to create machine-learning classifiers distinguishing four patient subgroups: patients with liver metastasis within or after 6 months; patients with metastasis at sites other than liver; and individuals who experienced no metastasis during the 3-year period of observation (Fig. 1). The result of the study underlines the power of utilising several different parameter types for achieving an accurate and – similarly important – robust classification of patients with a molecularly complex disease.^2^

The real importance of the work, however, lies not so much in the establishment of a biomarker panel. In fact, the study exhibits shortcomings in that respect, although performed in a prospective setting. Liver specimens from merely 49 PDAC patients and 19 non-cancerous controls were analysed. The small number may impede biomedical validity. Also, there is no validation of the classifiers. Verifying performance on independent cohorts of patients and control individuals is essential to establishing any biomarker signature.^3^ Finally, clinical data are only scarcely reported and information on comorbidities and adjuvant treatment is missing, leaving it unclear whether altered liver function (or systemic immune dysfunction) might have affected adjuvant therapy and thereby tumour recurrence.

The actual accomplishment of the study is the demonstration that focussing diagnostic approaches on the tumour only may obstruct the view on many other aspects that could be critical for dealing with cancer. In particular for metastasis, there are many biological processes beyond the biology of the tumour cells that are critical for their dissemination and the invasion of other organs. It has been shown that diverse processes take place that could be responsible and might be critical for creating a metastatic niche.^4^ It is therefore evident that the state of the tissue at which metastasis could occur has an influence on the actual process and could be utilised diagnostically. Selecting liver biopsies has another advantage. Besides being a primary site of PDAC metastasis, the organ is involved in countless biological processes throughout the body. This fact might be responsible for the ability of distinguishing patients, who will have metastases elsewhere in the body, from people without metastasis or early and late metastasis in the liver. In consequence, the paper is strongly advocating a wholistic look at a cancer disease.

This view at the system “cancer patient” rather than the pancreatic tumour and peritumoral tissues in isolation is likely to have clinical implications. Diagnostically, the work of Bojmar and colleagues suggests that it may be beneficial to take (biopsy) samples not only from the tumour but also from other tissues, with the liver probably being the most informative one. Disease monitoring and therapy could be adapted according to the results of this wider analysis. In addition, the described results confirm that combining different sample types and different molecular features is likely to be advantageous, in particular with respect to the robustness of a diagnostic classifier, once its performance has been firmly established. With cellular and molecular diagnostics being automated more and more by technical hardware developments and mediated by AI approaches, the assembly of the many pieces of information needed for such ends is not out of reach. Therapeutically, effector molecules might have a more specific effect when not targeting common features, such as proliferation. Influencing particularly the cells of a pre-metastatic niche in the liver, for example, may be superior in inhibiting metastasis and simultaneously act more specifically, thus avoiding detrimental side-effects.

In conclusion, the article by Bojmar et al. does not provide the one intriguing biomedical result that has direct consequences on the understanding, diagnostics or treatment of PDAC metastasis. However, it provides a variety of informative aspects that in combination make it a remarkable piece of scientific work: leads on relevant biological processes are provided; the potential is documented of utilising various molecular and cellular features in combination to achieve diagnostic relevance in predicting PDAC metastasis prior to the process actually taking place; and finally, and most importantly, it demonstrates that a systemic analysis of PDAC patients could reveal much more biomedically relevant information than a focus on the tumour alone.
